# Restless Legs Syndrome in Patients With *PMP22* ‐Related Neuropathies

**DOI:** 10.1111/jns.70123

**Published:** 2026-04-29

**Authors:** Bogdan Bjelica, Milorad Vujnic, Milica Vukojevic, Ivo Bozovic, Nikola Andrejic, Ivana Basta, Vidosava Rakocevic‐Stojanovic, Stojan Peric

**Affiliations:** ^1^ Department of Neurology Hannover Medical School Hannover Germany; ^2^ PRACTIS Clinician Scientist Program, Dean's Office for Academic Career Development Hannover Medical School Hannover Germany; ^3^ University of Belgrade Faculty of Medicine Belgrade Serbia; ^4^ Faculty of Medicine University of Banja Luka Banja Luka Republic of Srpska Bosnia and Herzegovina; ^5^ Neurology Clinic University Clinical Center of Serbia Belgrade Serbia

**Keywords:** Charcot–Marie–Tooth disease (CMT), hereditary neuropathy with liability to pressure palsies (HNPP), quality of life, restless legs syndrome (RLS)

## Abstract

**Background and Aims:**

Restless legs syndrome (RLS) is frequently reported in peripheral neuropathies, but its prevalence and clinical correlates in Charcot–Marie–Tooth disease type 1A (CMT1A) and hereditary neuropathy with liability to pressure palsies (HNPP) remain poorly defined. We aimed to determine RLS prevalence in CMT1A and HNPP and to assess associations with disease severity, muscle strength, disability, and quality of life (QoL).

**Methods:**

Forty‐seven CMT1A and 18 HNPP patients were included. RLS was diagnosed according to the International Restless Legs Syndrome Study Group criteria, and RLS severity was assessed with the International Restless Legs Syndrome Severity Scale (IRLS‐SS). MRC Sum Score (MRC‐SS), Charcot–Marie‐Tooth Examination Score (CMTES), Overall Neuropathy Limitations Scale (ONLS), Beck Depression Inventory (BDI), Fatigue Severity Scale (FSS), and the 36‐Item Short Form Health Survey (SF‐36) were recorded.

**Results:**

RLS was present in 29.8% of CMT1A and 38.9% of HNPP patients. CMT1A patients with RLS had longer disease duration (*p* = 0.05), worse muscle strength (*p* = 0.014), higher disease severity (*p* = 0.014), higher upper‐limb (*p* = 0.005) and overall disability (*p* = 0.011), and higher fatigue severity (*p* = 0.011) compared with those without RLS. HNPP patients with RLS showed higher upper‐limb (*p* = 0.034) and overall disability (*p* = 0.032), higher depression (*p* = 0.005), and fatigue severity (*p* = 0.018) than those without RLS. QoL was significantly impaired in patients with RLS in both groups, and RLS severity negatively correlated with physical and mental QoL domains.

**Interpretation:**

RLS is common in CMT1A and HNPP and is associated with increased disease severity, greater functional disability, and reduced QoL. Clinicians should screen for RLS in *PMP22*‐related neuropathies and consider symptomatic management.

## 1 Introduction

Hereditary polyneuropathies comprise a clinically and genetically diverse group of peripheral nervous system disorders caused by pathogenic variants in more than 100 genes [1]. The most prevalent subtype is Charcot–Marie–Tooth disease (CMT), which is further subdivided into demyelinating, axonal, and intermediate CMT [2]. Charcot–Marie–Tooth disease type 1A (CMT1A) and hereditary neuropathy with liability to pressure palsies (HNPP) represent two autosomal dominant, demyelinating neuropathies arising from mutations in the *PMP22* gene. A *PMP22* deletion causes HNPP, whereas a *PMP22* duplication results in CMT1A [2]. Although both disorders involve the same gene, their clinical manifestations differ substantially. CMT1A usually presents earlier, frequently includes musculoskeletal deformities, and is characterized by uniform demyelination and remyelination. In contrast, HNPP typically shows segmental demyelination and remyelination and is considered clinically milder than CMT1A [2, 3].

Restless legs syndrome (RLS) is a sensorimotor disorder characterized by an uncomfortable urge to move the legs that arises during periods of rest, improves transiently with movement or walking, typically intensifies at night, and can substantially impair quality of life (QoL) [4]. It has been linked to several neurological conditions, including Parkinson's disease, multiple sclerosis, amyotrophic lateral sclerosis, Alzheimer's dementia, and migraine [5, 6, 7, 8, 9]. RLS can also occur in individuals with peripheral neuropathies, although prevalence estimates vary considerably, ranging from 0% to 52.9% [10]. Several studies have examined RLS in patients with CMT [11, 12, 13, 14, 15], but these studies involved various sample sizes and heterogeneous CMT cohorts. Reported prevalence in demyelinating CMT also shows variability, from 0% in the study by Gemignani et al. to 40.9% [11] in the cohort studied by Boentert et al. [14]. Notably, no study to date has specifically focused on CMT1A and HNPP in assessing the occurrence of RLS, nor has any study systematically evaluated whether disease severity, functional disability, fatigue, or depressive symptoms are associated with RLS in these hereditary neuropathies.

The aim of our study was to assess the prevalence of RLS in a homogeneous cohort of CMT1A and HNPP patients and to analyze its association with muscle strength, disease severity, functional disability, fatigue, depressive symptoms, and QoL. Furthermore, we sought to analyze the predictors of RLS severity in CMT1A.

## 2 Materials and Methods

The study was approved by the Ethics Committee of the Faculty of Medicine, University of Belgrade, and the University Clinical Center of Serbia, and all participants provided written informed consent. We collected data from all patients diagnosed with CMT over a 10‐year period (2009–2018). All patients were diagnosed and treated at the Department for Neuromuscular Disorders, Neurology Clinic, University Clinical Centre of Serbia. Patients were eligible for inclusion if they were 18 years or older and had a confirmed duplication or deletion of the 17p11.2 chromosomal region containing the *PMP22* gene. Patients with significant comorbidities known to influence RLS (such as iron deficiency, chronic kidney disease, pregnancy, untreated hypothyroidism, severe anemia, or Parkinson's disease) were excluded. Following data were collected: sex, age at the time of testing, duration of the disease, use of walking aids, and presence of comorbid disorders. Copy number analysis of *PMP22* gene was conducted in the Genetic Laboratory of the Neurology Clinic, University Clinical Centre of Serbia, using real‐time quantitative polymerase chain reaction (RT‐PCR) with the TaqMan assay on an ABI 7500 Fast system (Applied Biosystems, USA). The HSA gene served as the endogenous control. All samples were analyzed in triplicate in independent reactions, and data were processed using the ΔΔ*Ct* method [16].

### 2.1 Assessment of Muscle Strength, Disease Severity and Functional Disability

Muscle strength was evaluated using the Medical Research Council (MRC) scale (ranging from zero to five), where zero indicates no muscle contraction and five reflects normal strength [17]. The total MRC Sum Score (MRC‐SS) was calculated by summing bilateral assessments of the following muscle groups: shoulder abductors, elbow flexors, wrist extensors, hip flexors, knee extensors, and foot dorsiflexors. Disease severity was assessed with the Charcot–Marie–Tooth Neuropathy Score (CMTNS), or with the CMT Examination Score (CMTES) when neurophysiological data were unavailable [18]. Neurophysiological studies were available for 27 CMT1A and 10 HNPP patients, while CMTES was obtained for all participants. Disease severity was classified as mild (CMTNS ≤ 10), moderate (CMTNS 11–20), or severe (CMTNS > 20) [18]. Functional disability was evaluated using the Overall Neuropathy Limitations Scale (ONLS) [19], which assesses limitations in performing activities of daily living due to upper‐ and lower‐limb impairment. The total score ranges from 0 to 12, with higher scores indicating greater functional disability.

### 2.2 Restless Legs Syndrome Assessment

The presence of RLS was determined according to the International Restless Legs Syndrome Study Group criteria through standardized physician interviews [20]. Patients who met these criteria completed the Serbian version of the International Restless Legs Syndrome Severity Scale (IRLS‐SS) [21], which assesses the severity of RLS symptoms and their impact on daily functioning. The IRLS‐SS consists of 10 items that evaluate limb discomfort, motor restlessness, symptom relief with movement, sleep disturbance, daytime sleepiness and fatigue, the severity and frequency of symptoms, the daily duration of symptoms, and the impact of RLS on daily activities and mood. The total score ranges from 0 to 40, with higher scores indicating more severe symptoms and greater disability. Severity categories were defined as follows: mild (0–10), moderate [11, 12, 13, 14, 15, 16, 17, 18, 19, 20], severe [21, 22, 23, 24, 25, 26, 27, 28, 29, 30], and very severe [21].

### 2.3 Assessment of Depression, Neuropathic Pain, Fatigue, Fear of Falling and Quality of Life

Depression was evaluated using the Beck Depression Inventory (BDI) [22]. A score of 11 or higher was considered indicative of depression. Fatigue was assessed with Krupp's Fatigue Severity Scale (FSS) [23], with scores above 36 on the nine‐item scale indicating clinically significant fatigue. Patients' concerns about falling were measured using the Falls Efficacy Scale (FES) [24]. This 10‐item questionnaire evaluates the degree of concern individuals experience regarding the possibility of falling during common daily activities, such as bathing, reaching for objects, or walking inside the home. A total score greater than 70 was considered a fear of falling [24].

A diagnosis of neuropathic pain was established according to the criteria of the International Association for the Study of Pain (IASP) [25]. Furthermore, patients were assessed using the painDETECT questionnaire (PD‐Q) [26]. The PD‐Q is a self‐report instrument comprising nine items, including seven weighted sensory pain descriptors and two items addressing the spatial (pain radiation or spreading) and temporal characteristics of the individual pain pattern. Patients were classified as having neuropathic pain if the PD‐Q score was ≥ 19. Furthermore, this group also included patients who were receiving neuropathic pain medication at the time of assessment and who were retrospectively evaluated as having neuropathic pain at the time of initiation of neuropathic pain treatment.

The Serbian version of the 36‐Item Short Form Health Survey (SF‐36) questionnaire was used to assess health‐related QoL [27]. This self‐report instrument encompasses eight domains: physical functioning (PF), role physical (RP), bodily pain (BP), general health (GH), vitality (VT), social functioning (SF), role emotional (RE), and mental health (MH). Furthermore, the Physical Composite Score (PCS), Mental Composite Score (MCS), and the total SF‐36 score as summary measures were calculated. All scores range from zero to 100, with higher values indicating better QoL.

### 2.4 Statistical Analysis

Statistical analyses were conducted using IBM SPSS Statistics (version 30; IBM Corp., Armonk, NY, USA). Data normality was assessed with the Shapiro–Wilk and Kolmogorov–Smirnov tests as well as graphical inspection of histograms. Continuous variables were reported as mean and standard deviation (SD). Group comparisons were performed using the *χ* ^2^ test, Fisher's exact test, Mann–Whitney *U* test, or Student's *T*‐test, as appropriate. A multiple linear regression analysis (enter method) was performed with the IRLS‐SS total score as the dependent variable in order to assess the predictors of RLS severity. This analysis was performed only in CMT1A patients due to the small sample size of our HNPP cohort. Multicollinearity among independent variables was assessed using variance inflation factors (VIFs). To avoid overfitting in the context of a small sample size, the multivariable regression model was restricted to key clinically relevant variables. Age and sex were included as covariates. A *p*‐value < 0.05 was considered statistically significant, while *p* < 0.01 indicated high statistical significance.

## 3 Results

### 3.1 Restless Legs Syndrome in CMT1A Patients

Fifty‐one patients with CMT1A were initially included. Four patients were excluded: one patient had iron deficiency and diabetes mellitus; another had chronic obstructive pulmonary disease and diabetes mellitus; and the third and fourth patients had diabetes mellitus. Therefore, the final CMT1A cohort consisted of 47 participants.

Nearly one‐third of patients with CMT1A (*n* = 14, 29.8%) had RLS. Main sociodemographic and clinical characteristics of CMT1A patients with and without RLS are presented in Table 1. No significant differences were observed between the two groups with respect to sex, age, or the presence of neuropathic pain, nor regarding current use of neuropathic pain medication (*p* > 0.05). None of the patients were receiving dopaminergic therapy for RLS. CMT1A patients with RLS had a longer disease duration (28.1 ± 16.3 vs. 18.1 ± 15.1 years, *p* = 0.05), lower muscle strength (46.5 ± 7.0 vs. 51.5 ± 5.7, *p* = 0.014), higher CMTES scores (14.1 ± 5.6 vs. 9.8 ± 5.2, *p* = 0.014), higher upper‐limb ONLS score (2.1 ± 0.9 vs. 1.1 ± 0.8, *p* = 0.005) and total ONLS score (4.4 ± 1.6 vs. 3.0 ± 1.6, *p* = 0.011) and were more likely to use walking aids (57.1% vs. 24.2%, *p* = 0.045) compared with CMT1A patients without RLS. Furthermore, CMT1A patients with RLS had higher FSS scores (50.6 ± 17.5 vs. 34.4 ± 19.7, *p* = 0.011), and higher FES scores (40.8 ± 20.6 vs. 23.8 ± 21.6, *p* = 0.016) compared with CMT1A patients without RLS.

**TABLE 1:** Main sociodemographic and clinical characteristics of CMT1A patients with and without RLS.

Features	Without RLS	With RLS	*p*
*N* (%)	33 (70.2%)	14 (29.8%)	
Sex (% of males)	41.4%	35.7%	0.753
Age at the time of testing (years, mean ± SD)	48.5 ± 13.9	55.2 ± 10.8	0.112
Disease duration (years, mean ± SD)	18.1 ± 15.1	28.1 ± 16.3	**0.050**
% of patients using walking aids (%)	24.2%	57.1%	**0.045**
Presence of at least one comorbidity (%)^a^	53.1%	57.1%	1.000
Presence of neuropathic pain (%)	28.1%	26.7%	1.000
Currently on neuropathic pain medication (%)^b^	21.9%	13.3%	0.697
MRC‐SS score (mean ± SD)	51.5 ± 5.7	46.5 ± 7.0	**0.014**
CMTNS score (mean ± SD)	14.7 ± 6.0	17.9 ± 6.8	0.235
CMTES score (mean ± SD)	9.8 ± 5.2	14.1 ± 5.6	**0.014**
Disease severity (according to CMTNS) (%)			
Mild	35.0%	12.5%	
Moderate	55.0%	50.0%	
Severe	10.0%	37.5%	0.177
ONLS score (mean ± SD)			
Upper limbs	1.1 ± 0.8	2.1 ± 0.9	**0.005**
Lower limbs	1.9 ± 0.8	2.3 ± 0.8	0.115
Total	3.0 ± 1.6	4.4 ± 1.6	**0.011**
MMSE score (mean ± SD)	28.2 ± 1.8	27.3 ± 2.8	0.223
BDI score (mean ± SD)	7.1 ± 8.7	11.4 ± 11.6	0.169
% of depressed	21.9%	35.7%	0.467
FSS score (mean ± SD)	34.4 ± 19.7	50.6 ± 17.5	**0.011**
% of fatigued	45.5%	78.6%	0.055
FES score (mean ± SD)	23.8 ± 21.6	40.8 ± 20.6	**0.016**
% of patients with fear of falling	6.1%	7.1%	1.000

*Note:* Bold values indicate statistical significance (*p* < 0.05).

Abbreviations: BDI, Beck Depression Inventory; CMTES, Charcot–Marie–Tooth Examination Score; CMTNS, Charcot–Marie–Tooth Neuropathy Score; FES, Falls Efficacy Score; FSS, Fatigue Severity Scale; MMSE, Mini‐Mental State Examination; MRC‐SS, Medical Research Council Sum Score; *N*, number; ONLS, Overall Neuropathy Limitations Scale; RLS, restless legs syndrome; SD, standard deviation.

^a^ Comorbidities among CMT1A patients with RLS included hypercholesterolemia in four patients, hypertension in three, and cardiac arrhythmia in two. Prostate hyperplasia, Reiter's syndrome, mitral valve prolapse, glaucoma, renal lithiasis, migraine, and depression were noted in single cases. Among CMT1A patients without RLS, three had hypertension and hypercholesterolemia, and single cases of retinal ablation and osteoporosis were observed.

^b^ Eleven patients received pregabalin and one patient amitriptyline.

### 3.2 RLS Severity and Its Predictors in CMT1A Patients

The total IRLS‐SS score in patients with CMT1A and RLS was 21.6 ± 8.4. Patients with CMT1A and RLS most frequently reported a very severe feeling of discomfort due to RLS (41.2%). About two‐thirds of patients with CMT1A and RLS reported moderate to severe RLS symptoms, 64.7% had sleep disturbances due to RLS, and slightly more than half (52.9%) felt tired and sleepy during the day because of RLS. RLS occurred in 88.2% of patients two or more days per week, while one‐third of the patients experienced RLS six or seven days per week.

In the unilateral analysis, age, sex, and disease duration were not significantly associated with RLS severity (all *p* > 0.05). Multiple linear regression analysis (enter method) with RLS severity (IRLS‐SS) as the dependent variable is shown in Table 2. The overall model was statistically significant (*F* = 5.63, *p* = 0.012), explaining 56.9% of the variance in IRLS‐SS (adjusted *R* ^2^ = 0.569). Higher BDI scores (*B* = 0.53, *p* = 0.004) and higher CMTES scores (*B* = 0.81, *p* = 0.024) were independently associated with higher IRLS‐SS.

**TABLE 2:** Multiple regression analysis with RLS severity as dependent variable (enter method).

Predictor	*B*	SE	*β*	*t*	*p*	95% CI
Sex	−5.16	3.56	−0.30	−1.45	0.178	[−13.1–2.8]
Age at the time of testing	−0.16	0.14	−0.21	−1.14	0.282	[−0.5–0.2]
CMTES	0.81	0.30	0.52	2.66	**0.024**	[0.1–1.5]
BDI	0.53	0.14	0.71	3.67	**0.004**	[0.2–0.8]

*Note:* Bold values indicate statistical significance (*p* < 0.05).

Abbreviations: 95% CI, 95% confidence interval; *β*, standardized regression coefficient; *B*, unstandardized regression coefficient; BDI, Beck Depression Inventory; CMTES, Charcot–Marie–Tooth Examination Score; *p*, significance level; SE, standard error; *t*, t‐statistic.

### 3.3 Quality of Life in CMT1A Patients With and Without Restless Legs Syndrome

**FIGURE 1:**
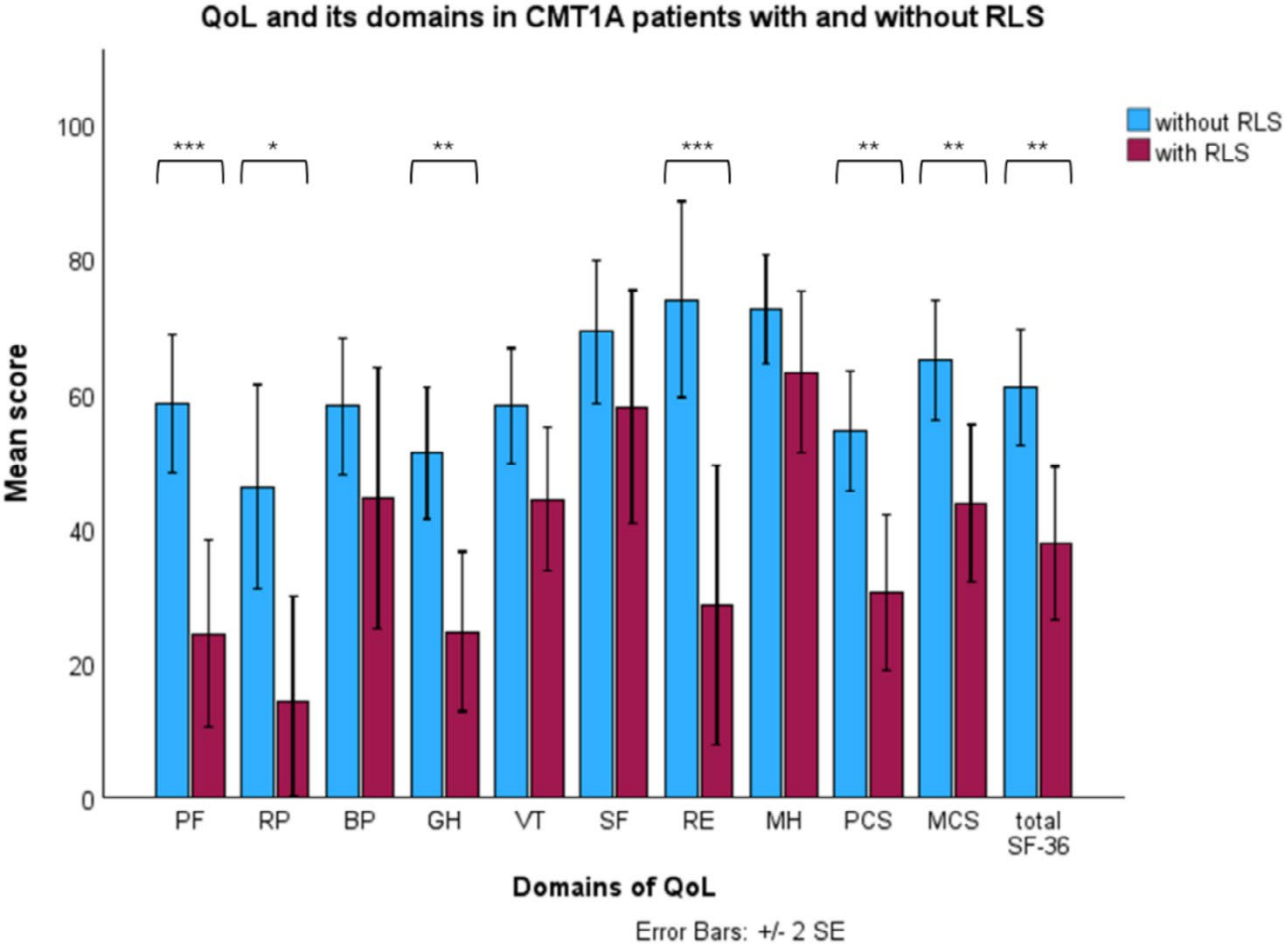
Quality of life and its domains in CMT1A patients with and without restless legs syndrome. BP, bodily pain; CMT1A, Charcot–Marie–Tooth disease type 1A; GH, general health; MCS, mental composite score; MH, mental health; PCS, physical composite score; PF, physical functioning; QoL, quality of life; RE, role emotional; RLS, restless legs syndrome; RP, role physical; SE, standard error; SF, social functioning; SF‐36, 36‐Item Short Form Health Survey; VT, vitality. *p < 0.05; **p < 0.01; ***p < 0.001.

Figure 1 shows QoL and its domains in CMT1A patients with and without RLS. CMT1A patients with RLS had worse overall QoL (37.7 ± 21.4 vs. 60.9 ± 24.5, *p* = 0.004) and worse scores in the following domains of QoL, compared to CMT1A patients without RLS: physical functioning (24.1 ± 25.6 vs. 58.4 ± 28.9, *p* < 0.001), role physical (14.3 ± 28.9 vs. 46.1 ± 43.1, *p* = 0.016), general health (24.6 ± 22.2 vs. 51.1 ± 27.6, *p* = 0.003), role emotional (28.6 ± 38.9 vs. 73.9 ± 41.2, *p* = 0.001), physical composite score (30.4 ± 21.6 vs. 54.4 ± 25.2, *p* = 0.003), and mental composite score (43.7 ± 21.8 vs. 64.9 ± 25.2, *p* = 0.009). RLS severity (IRLS‐SS) showed a moderate negative correlation with the overall QoL (Figure S1; *ρ* = −0.65, *p* = 0.013).

### 3.4 Restless Legs Syndrome in HNPP Patients

A total of 18 patients were included in the study examining RLS in patients with HNPP. Slightly more than one‐third of the patients (*n* = 7, 38.9%) with HNPP had RLS. Their detailed sociodemographic and clinical data are presented in Table 3. No differences in sex or age were observed between the two groups (*p* > 0.05). Patients with HNPP and RLS had greater upper limb (1.6 ± 1.0 vs. 0.6 ± 0.8, *p* = 0.034) and overall disability as measures with ONLS (2.9 ± 1.6 vs. 1.2 ± 1.7, *p* = 0.032), as well as higher BDI scores (15.1 ± 12.6 vs. 4.4 ± 9.4, *p* = 0.005), higher FSS scores (40.9 ± 20.7 vs. 19.2 ± 16.9, *p* = 0.018), and higher FES scores (29.4 ± 13.9 vs. 14.6 ± 14.7, *p* = 0.035) compared to HNPP patients without RLS.

**TABLE 3:** Main sociodemographic and clinical characteristics of HNPP patients with and without RLS.

Features	Without RLS	With RLS	*p*
*N*	11	7	
Sex (% of males)	54.5%	42.9%	1.000
Age at the time of testing (years, mean ± SD)	39.8 ± 15.7	41.9 ± 15.1	0.789
Disease duration (years, mean ± SD)	15.2 ± 11.4	14.9 ± 13.2	0.954
% of patients using walking aids (%)	0.0%	0.0%	—
Presence of at least one comorbidity (%)^a^	63.6%	71.4%	1.000
Presence of neuropathic pain (%)	9.1%	57.1%	**0.047**
Currently on neuropathic pain medication (%)^b^	9.1%	28.6%	0.537
MRC‐SS score (mean ± SD)	56.3 ± 5.6	52.9 ± 5.5	0.220
CMTNS score (mean ± SD)	7.4 ± 4.8	9.0 ± 2.8	0.680
CMTES score (mean ± SD)	6.2 ± 4.3	10.3 ± 4.0	0.059
Disease severity (according to CMTNS) (%)			
Mild	71.4%	50.0%	
Moderate	28.6%	50.0%	
Severe	0.0%	0.0%	1.000
ONLS score (mean ± SD)			
Upper limbs	0.6 ± 0.8	1.6 ± 1.0	**0.029**
Lower limbs	0.6 ± 1.0	1.3 ± 0.8	0.170
Total	1.2 ± 1.7	2.9 ± 1.6	**0.032**
MMSE score (mean ± SD)	29.1 ± 1.1	29.2 ± 0.4	0.974
BDI score (mean ± SD)	4.4 ± 9.4	15.1 ± 12.6	**0.005**
% of depressed	9.1%	42.9%	0.245
FSS score (mean ± SD)	19.2 ± 16.9	40.9 ± 20.7	**0.018**
% of fatigued	9.1%	42.9%	0.245
FES score (mean ± SD)	14.6 ± 14.7	29.4 ± 13.9	**0.035**
% of patients with fear of falling	0.0%	0.0%	—

*Note:* Bold values indicate statistical significance (*p* < 0.05).

Abbreviations: BDI, Beck Depression Inventory; CMTES, Charcot–Marie–Tooth Examination Score; CMTNS, Charcot–Marie–Tooth Neuropathy Scale; FES, Falls Efficacy Score; FSS, Fatigue Severity Scale; MMSE, Mini Mental State Examination; MRC‐SS, Medical Research Council Sum Score; N, number; ONLS, Overall Neuropathy Limitation Scale; RLS, restless legs syndrome; SD, standard deviation.

^a^ Comorbidities among HNPP patients with RLS included hypertension and Hashimoto thyroiditis (euthyroid at the moment of testing) in two patients. Angina pectoris, myocardial infarction, radiculopathy, celiac disease, spondylosis, previous history of breast cancer, cardiac arrythmia, hyperlipidemia, asthma and chronic sinusitis were noted in single cases. Among HNPP patients without RLS, three had hypertension, two had ulcerative colitis, and single cases of cardiac arrhythmia, asthma, previous history of thyroid cancer, brain aneurysm and gastric ulcer were observed.

^b^ All patients were on pregabalin.

The total RLS score in patients with HNPP and RLS was 18.0 ± 5.5. Patients with HNPP and RLS experienced mild (42.9%) or moderate (57.1%) discomfort in the limbs due to RLS. Most patients (71.4%) with HNPP reported complete or almost complete relief of RLS symptoms when walking. About two‐thirds of patients with HNPP and RLS reported moderate to severe RLS symptoms, 64.7% had sleep disturbances due to RLS, and slightly more than half (52.9%) had daytime sleepiness because of RLS. RLS occurred in 88.2% of patients two or more days per week, while one‐third of the patients experienced RLS six or seven days per week.

### 3.5 Quality of Life in HNPP Patients With and Without Restless Legs Syndrome

**FIGURE 2:**
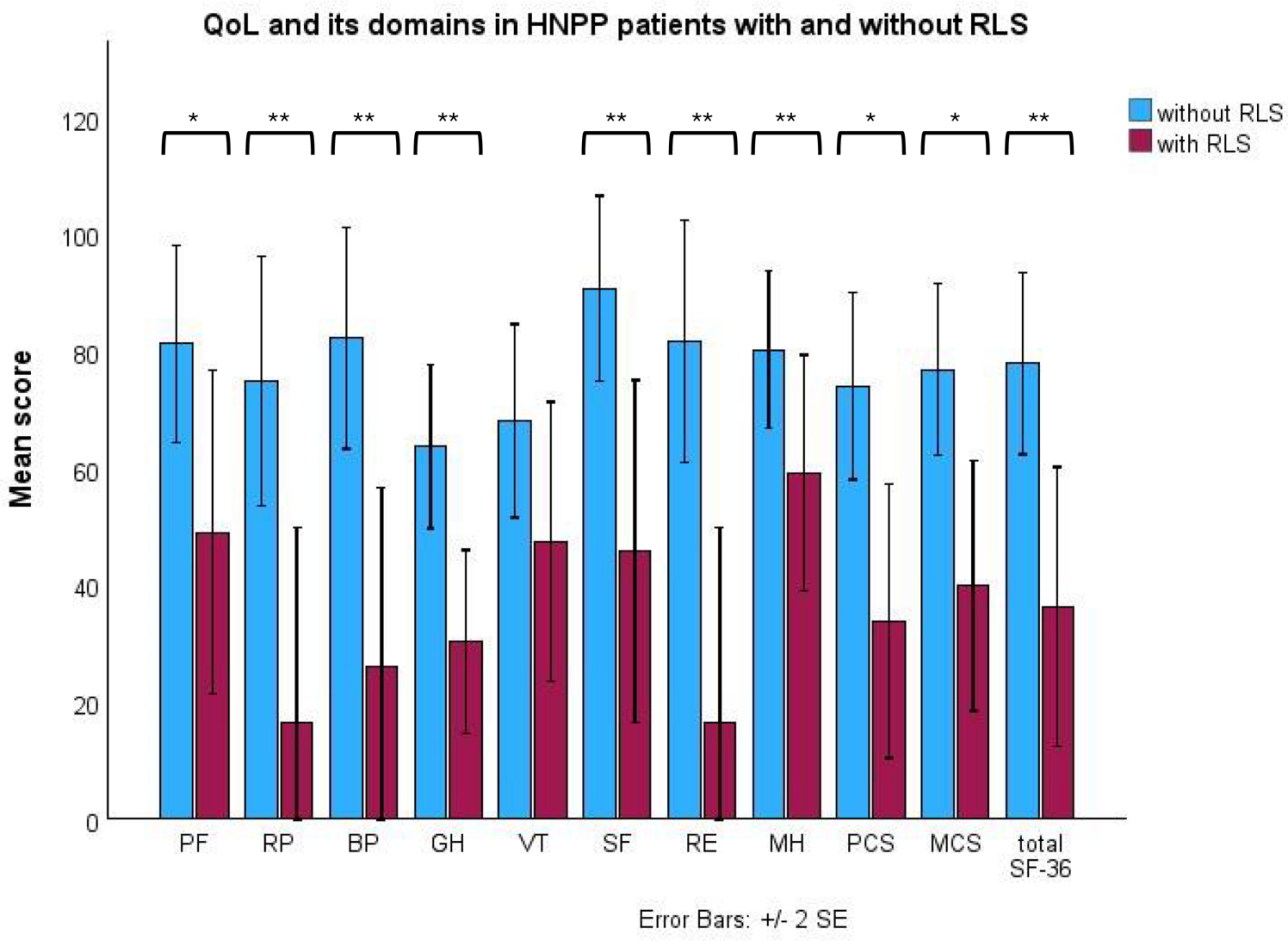
Quality of life and its domains in HNPP patients with and without restless legs syndrome. BP, bodily pain; GH, general health; HNPP, hereditary neuropathy with liability to pressure palsies; MCS, mental composite score; MH, mental health; PCS, physical composite score; PF, physical functioning; QoL, quality of life; RLS, restless legs syndrome; RP, role physical; RE, role emotional; SE, standard error; SF, social functioning; SF‐36, 36‐Item Short Form Health Survey; VT, vitality. *p < 0.05; **p < 0.01.

Figure 2 shows the QoL and its domains in HNPP patients with and without RLS. HNPP patients with RLS had significantly worse scores in all QoL domains, except in vitality, compared to HNPP patients without RLS: physical functioning (45.7 ± 32.2 vs. 81.4 ± 27.9, *p* = 0.024), role physical (16.7 ± 40.8 vs. 75.0 ± 35.5, *p* = 0.008), bodily pain (26.0 ± 37.6 vs. 82.3 ± 31.4, *p* = 0.005), general health (27.4 ± 19.2 vs. 63.8 ± 23.2, *p* = 0.003), social functioning (45.8 ± 35.9 vs. 90.9 ± 26.3, *p* = 0.009), role emotional (16.7 ± 40.8 vs. 81.8 ± 34.5, *p* = 0.003), mental health (52.6 ± 28.8 vs. 80.4 ± 22.4, *p* = 0.035), as well as mental (39.9 ± 26.2 vs. 77.0 ± 24.3, *p* = 0.01) and physical composite score (33.9 ± 28.7 vs. 74.1 ± 26.5, *p* = 0.011). Furthermore, HNPP patients with RLS had significantly worse overall QoL, compared to HNPP patients without RLS (36.4 ± 29.2 vs. 78.0 ± 25.7, *p* = 0.008). RLS severity (IRLS‐SS) showed no correlation with overall QoL in HNPP patients with RLS.

## 4 Discussion

To the best of our knowledge, our study provides the first description of RLS in a homogeneous cohort of patients with CMT1A and HNPP and examines its association with muscle strength, disease severity, functional disability, and depressive symptoms. RLS was present in nearly one‐third of individuals with CMT1A and in more than one‐third of those with HNPP. This represents a markedly higher prevalence compared to the adult general population, where RLS affects approximately 5%–13% of individuals [4]. Our findings are consistent with a recently published meta‐analysis reporting that the prevalence of RLS in patients with peripheral neuropathies ranges from 5.2% to 53.7%, with a pooled prevalence of 21.5% across seven studies [10]. With respect to CMT specifically, our study is in line with previous studies that reported that 28% (*n* = 14; [13]) and 22% of CMT patients had RLS [12]. Slightly higher prevalence has been reported in a previous study involving 61 CMT1 patients (34 CMT1A) [14]. Contrary to this, Gemignani et al. reported that none of their 17 CMT1 patients had RLS [11]. Regarding HNPP, our results differ from those of Luigetti et al. [13], who found no meaningful association between HNPP and RLS, identifying RLS in only 1 of 15 HNPP patients.

We further examined the relationship between the presence of RLS and sociodemographic as well as clinical characteristics in our cohort. The presence of RLS in our CMT1A patients was associated with greater muscle weakness, higher functional disability, and greater disease severity. In our HNPP patients, the presence of RLS was associated with higher disability, but not with muscle weakness or disease severity. To date, no study has examined the relationship between muscle strength, disability, and disease severity with RLS specifically in CMT1A or HNPP. In chronic inflammatory demyelinating polyneuropathy, an association between RLS and lower limb weakness and functional disability has been reported [27]. Although CMT1A and HNPP are primarily demyelinating neuropathies, both conditions are characterized by secondary and progressive axonal loss [2, 28]. Compound muscle action potential amplitude and the degree of axonal loss are known to correlate with disease progression, disease severity, and disability in CMT1A [28, 29]. Previous studies have also linked RLS to axonal loss and atrophy [11, 30]. Taken together, these findings support the possibility that RLS in CMT1A and HNPP may arise as a consequence of secondary axonal degeneration. Furthermore, it would be of interest to independently evaluate electrophysiological parameters in relation to RLS severity. Previous studies have reported associations between RLS and specific electrophysiological measures, such as *F*‐wave duration/compound muscle action potential amplitude ratios in the upper and lower limbs and sympathetic skin responses [31, 32].

Our patients with CMT1A and RLS had, on average, a disease duration approximately 10 years longer than those without RLS. This raises the possibility that some CMT1A patients without RLS at the time of assessment may develop RLS later in the disease course. Given the cross‐sectional design of the study, this may have resulted in an underestimation of the true lifetime prevalence of RLS in CMT1A. In contrast, no such association between disease duration and RLS was observed in HNPP, which may reflect differences in cumulative peripheral nerve involvement and disease progression between the two neuropathies. Longitudinal studies are therefore needed to clarify the temporal relationship between neuropathy progression and the development of RLS.

Our CMT1A and HNPP patients with RLS demonstrated poorer overall QoL compared to those without RLS. Among CMT1A patients with RLS, the lowest scores were observed in the role functioning, physical functioning, and general health domains, while the highest scores were seen in mental health and social functioning. To the best of our knowledge, only one study has examined the relationship between QoL and the presence of RLS in a web‐based survey of a heterogeneous, self‐reported CMT cohort [12]. However, this study did not directly compare QoL or its specific domains between patients with and without RLS. Consistent with our findings, it reported a negative correlation between RLS severity and both the physical and mental components of QoL, and also identified RLS severity as an independent predictor of the physical composite score [12]. In our cohort, RLS severity showed a significant negative association with physical and mental QoL domains as well as overall QoL in CMT1A patients, but this relationship was not observed in HNPP patients. Neurologists should be aware of the occurrence of RLS in *PMP22*‐related neuropathies and consider offering appropriate symptomatic treatment.

The main limitations of our study include the absence of a control group and its monocentric and cross‐sectional design. In addition, we did not incorporate other subjective measures (e.g., Pittsburgh Sleep Quality Index, Epworth Sleepiness Scale) or objective assessments such as overnight polysomnography, which would have allowed for a more comprehensive characterization of RLS as a sleep disorder. Furthermore, we did not systematically collect data on the age at onset of RLS in our patients, which would have provided additional insight into the interval between the clinical onset of neuropathy and RLS. Future studies should address these limitations in larger cohorts and employ a multicenter design.

In conclusion, RLS is common in both CMT1A and HNPP. In CMT1A, its presence was associated with greater functional disability and disease severity, while in HNPP it was linked to higher disability. Both the presence and severity of RLS negatively impacted QoL, highlighting the importance for neurologists to recognize RLS in *PMP22*‐related neuropathies and to consider appropriate symptomatic management.

## Supporting information


**Figure S1:** Correlation between the restless legs syndrome severity and the QoL in patients with CMT1A
RLS, restless legs syndrome; QoL, quality of life; CMT1A, Charcot–Marie–Tooth type 1A; IRLS‐SS, International Restless Legs Syndrome Severity Scale; SF‐36, 36‐Item Short Form Health Survey.

## Data Availability

The data that support the findings of this study are available from the corresponding author upon reasonable request.
