# Supplementary figures and images for: Restless Legs Syndrome in Patients With PMP22 ‐Related Neuropathies
Source: J Peripher Nerv Syst. 2026 Apr 29;31:e70123. doi: 10.1111/jns.70123 (PMC13126250; doi:10.1111/jns.70123)

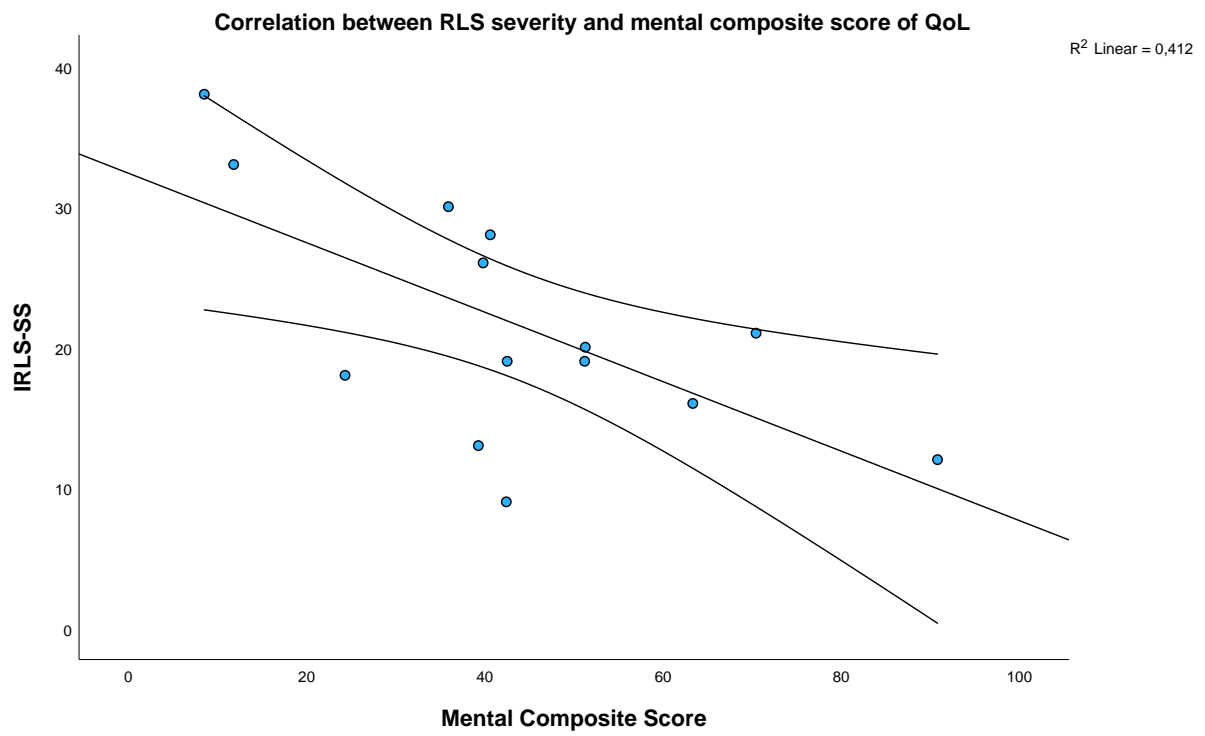

Supplement: Supplementary file 1 — Figure S1: Correlation between the restless legs syndrome severity and the QoL in patients with CMT1A RLS, restless legs syndrome; QoL, quality of life; CMT1A, Charcot–Marie–Tooth type 1A; IRLS‐SS, International Restless Legs Syndrome Severity Scale; SF‐36, 36‐Item Short Form Health Survey. [file JNS-31-0-s001.zip › jns70123-sup-0001-Supplementary_Figure_S1A.pdf]

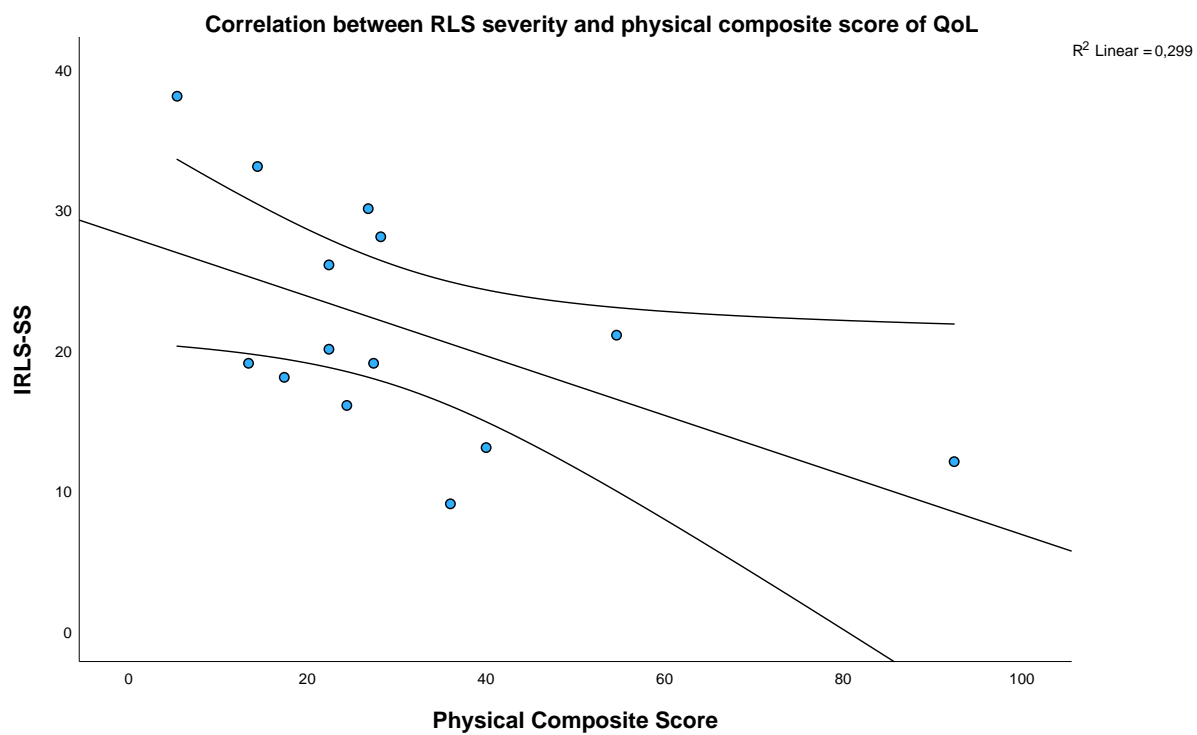

Supplement: Supplementary file 1 — Figure S1: Correlation between the restless legs syndrome severity and the QoL in patients with CMT1A RLS, restless legs syndrome; QoL, quality of life; CMT1A, Charcot–Marie–Tooth type 1A; IRLS‐SS, International Restless Legs Syndrome Severity Scale; SF‐36, 36‐Item Short Form Health Survey. [file JNS-31-0-s001.zip › jns70123-sup-0002-Supplementary_Figure_S1B.pdf]

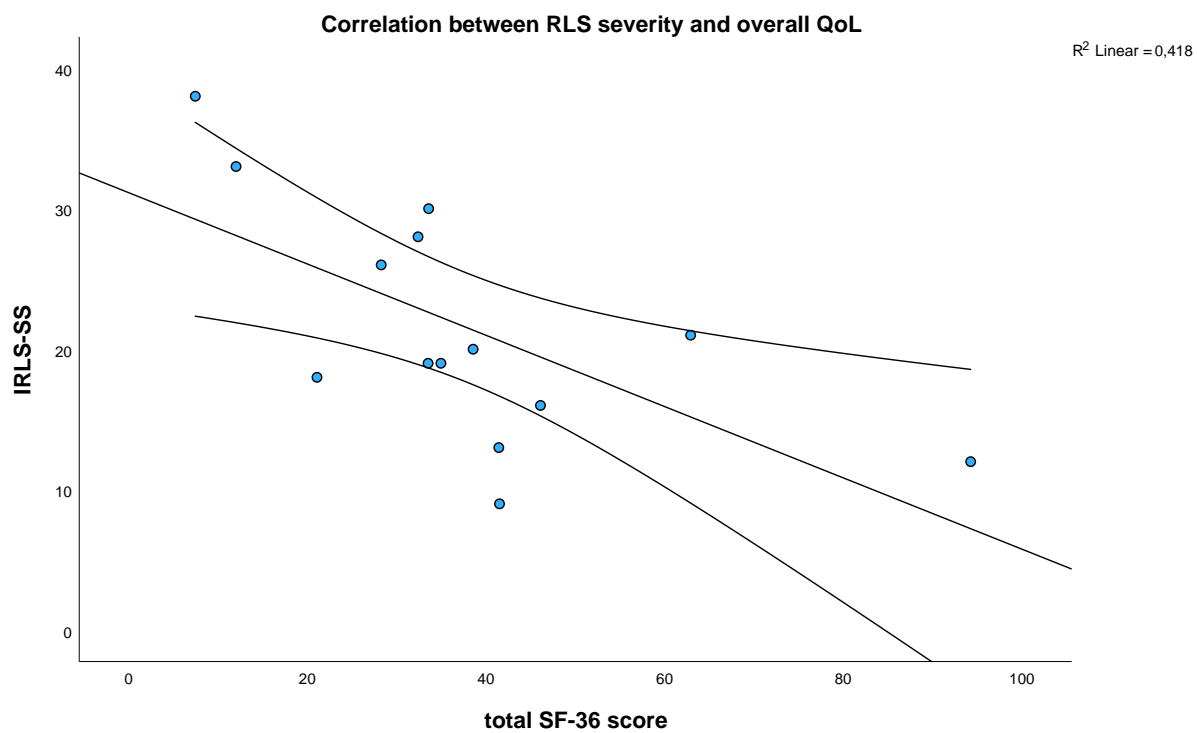

Supplement: Supplementary file 1 — Figure S1: Correlation between the restless legs syndrome severity and the QoL in patients with CMT1A RLS, restless legs syndrome; QoL, quality of life; CMT1A, Charcot–Marie–Tooth type 1A; IRLS‐SS, International Restless Legs Syndrome Severity Scale; SF‐36, 36‐Item Short Form Health Survey. [file JNS-31-0-s001.zip › jns70123-sup-0003-Supplementary_Figure_S1C.pdf]
